# Supplementary material for: Single cell multi-omic analysis identifies a Tbx1-dependent multilineage primed population in murine cardiopharyngeal mesoderm
Source: Nat Commun. 2021 Nov 17;12:6645. doi: 10.1038/s41467-021-26966-6 (PMC8599455; doi:10.1038/s41467-021-26966-6)
Supplement: Supplementary file 1 — Supplementary Information [file 41467_2021_26966_MOESM1_ESM.pdf]

Supplementary Table 1. Sorted cell number for scRNA-seq

Time point	cell number (submitted)
Mesp1^{Cre}; *ROSA26-GFP*$^{f/+}$	
E8.0	16,416
E8.25	8,364
E9.5	12,000
E9.5	16,000
E10.5	18,038
Mesp1^{Cre};*ROSA26-GFP*$^{f/+}$; *Tbx1*$^{f/f}$	
E9.5	13,788
E9.5	16,000
Tbx1$^{Cre/+}$; *ROSA26-GFP*$^{f/+}$	
E8.5	4,975
E9.5	18,300
Tbx1$^{Cre/f}$; *ROSA26-GFP*$^{f/+}$	
E8.5	2,820
E9.5	11,560

Supplementary Table 1. Sorted cell number for scRNA-seq. Cell number submitted to the Chromium 10x controller for scRNA-seq. This is additional information for Table 1.

Supplementary Table 2: RNAscope probes		
RNAscope® Probe - Mm-Aplnr	Advanced Cell Diagnostics	Cat# 436171
RNAscope® Probe - Mm-Nrg1-C3	Advanced Cell Diagnostics	Cat# 418181-C3
RNAscope® Probe - Mm-Isl1-C2	Advanced Cell Diagnostics	Cat# 451931-C2
RNAscope® Probe -Mm-Tbx1	Advanced Cell Diagnostics	Cat# 481911
RNAscope® Probe -EGFP-C3	Advanced Cell Diagnostics	Cat# 400281-C3
RNAscope® Probe - Mm-Pax8-C2	Advanced Cell Diagnostics	Cat# 574431-C2
RNAscope® Probe - Mm-Sema3c-C3	Advanced Cell Diagnostics	Cat# 441441-C3

Supplementary Table 2. Gene probes used for RNAscope experiments. Catalog numbers for the RNAscope probes are provided.

Supplement figure 1

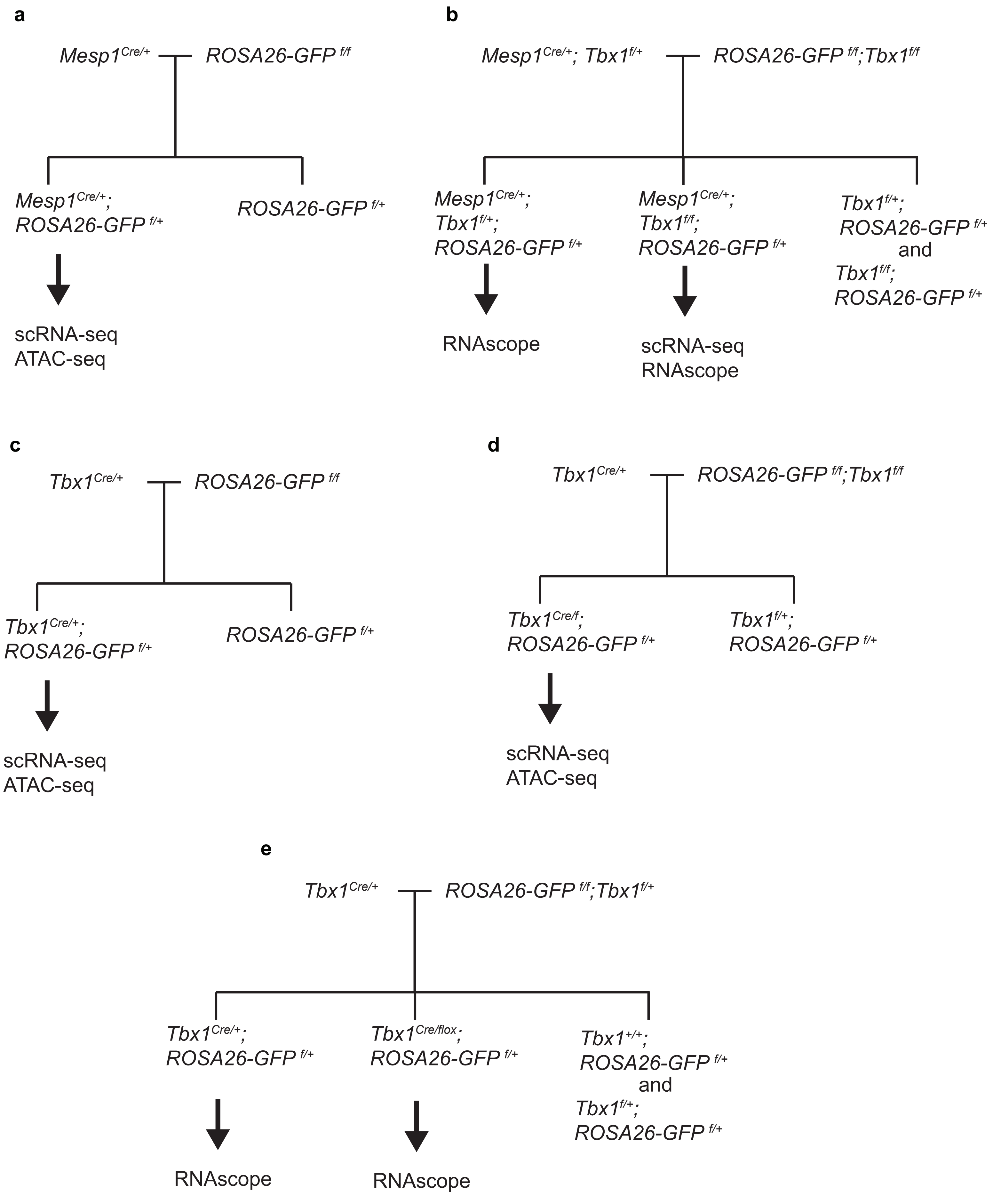

Supplementary Fig. 1. The mouse mating strategy for each experiment

a. Cross to perform lineage tracing using *Mesp1*$^{Cre/+}$ and *ROSA26-GFP*$^{f/f}$ mice. Genotypes of embryos used for individual experiments are indicated.

b. Cross to inactivate *Tbx1* in the *Mesp1*^{Cre} lineage using *ROSA26-GFP*$^{f/f}$ mice to trace the lineage is shown. Genotypes of embryos used for individual experiments are indicated. RNAscope analysis was performed with *Mesp1*$^{Cre/+}$*;Tbx1*$^{f/+}$ heterozygous control versus *Mesp1*$^{Cre/+}$*;Tbx1*$^{f/f}$ conditional null embryos from the same litter.

c. Cross to perform lineage tracing using *Tbx1*$^{Cre/+}$ and *ROSA26-GFP*$^{f/f}$ mice. Genotypes of embryos used for individual experiments are indicated.

d. Cross to inactivate *Tbx1* in the *Tbx1*^{Cre} lineage using *ROSA26-GFP*$^{f/f}$ mice to trace the lineage is shown. Genotypes of embryos used for individual experiments are indicated.

e. Crosses to inactivate *Tbx1* and perform RNAscope experiments are shown. *Tbx1*$^{Cre/+}$ embryos were compared with *Tbx1*$^{Cre/f}$ embryos from the same litter.

Supplement figure 2

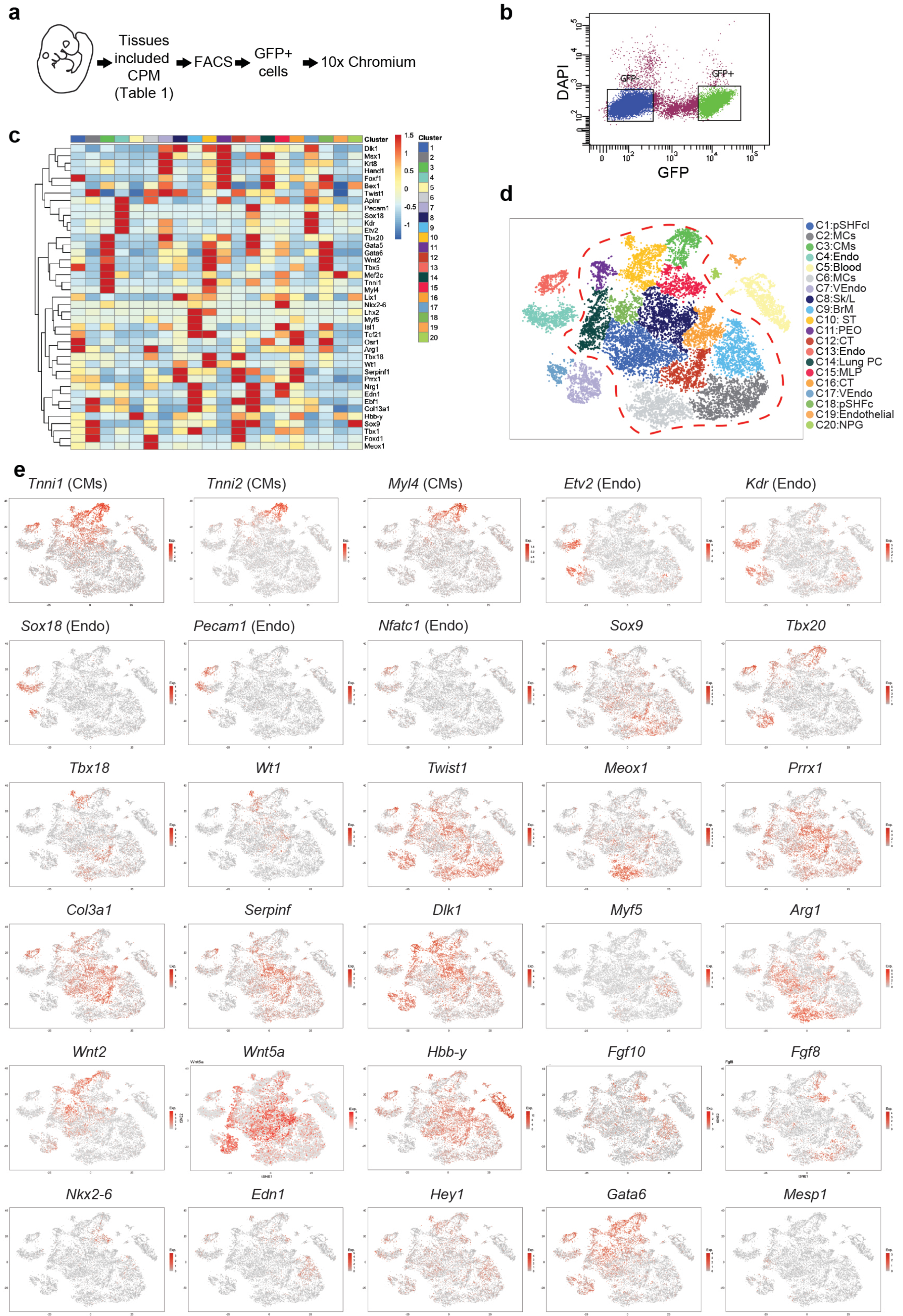

Supplementary Fig. 2. Sample preparation and cluster identification of *Mesp1*+ lineages at E8.0 to E10.5 for the scRNA-seq dataset

a. Scheme of sample preparation for scRNA-seq. After dissection of tissues from the embryo, GFP+ cells were sorted. Following cell counting with checking cell viability, cells were loaded to the 10x Chromium instrument.

b. The gating example of FACS purification. Only the GFP+, DAPI- population was collected for scRNA-seq.

c. Heatmap of average gene expression level of marker genes in all clusters of *Mesp1*+ lineages at E8.0 to E10.5 in the scRNA-seq dataset.

d. tSNE plot colored by clusters with the cluster selection information. Red dotted line represents the cluster group used for further analysis in Fig. 2a.

e. tSNE plot colored by expression level of marker genes. The color spectrum from gray to red indicates expression level from low to high.

Supplement figure 3

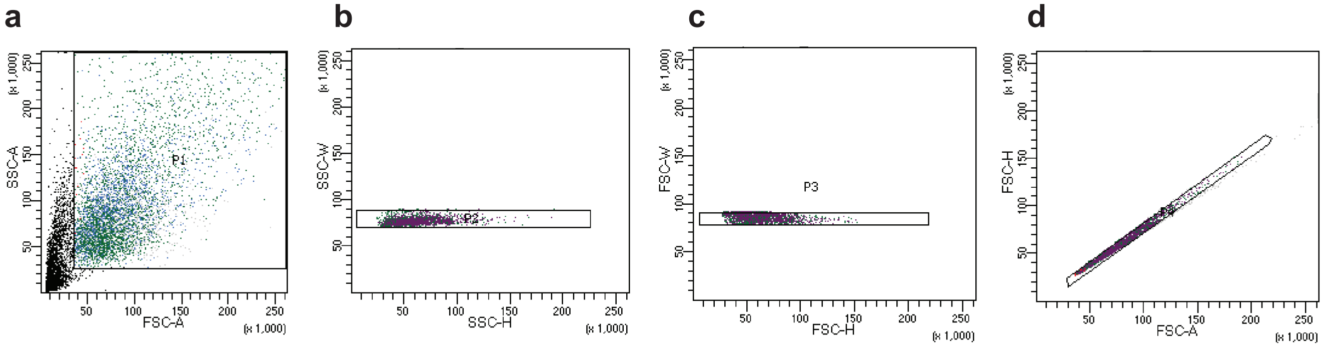

Supplementary Fig. 3. Gating strategy of GFP positive cell sorting (related to Supplementary Fig. 2b)

a. FSC-A/SSC-A (FSC-A, forward scatter area; SSC-A, side scatter area) plot. The cells in P1 were gated as cells.

b-d. The singlets were gated in SSC-H/SSC-W (SSC-height; SSC-width) plot (**b**) as P2, in FSC-H/FSC-W (FSC-height; FSC-width) plot (**c**) as P3

and in FSC-A/FSC-H plot (**d**) as P4 in order. The singlets in P4 gate were plotted as in Supplementary Fig. 2b, to sort GFP positive versus DAPI negative cells.

Supplement figure 4

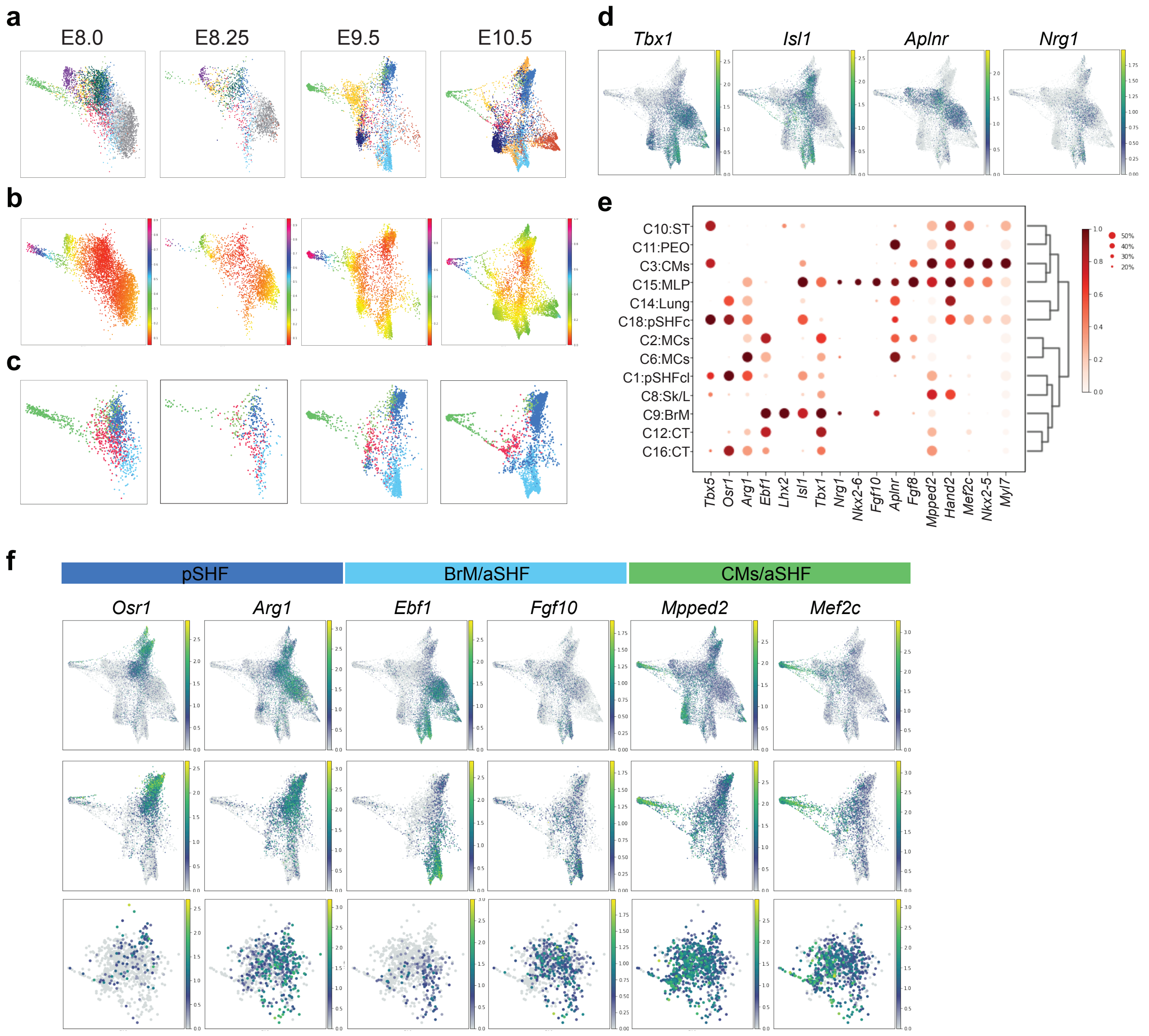

Supplementary Fig. 4. Gene expression on single-cell embedding graph of *Mesp1*+ lineages at E8.0 to E10.5 in the scRNA-seq dataset

a. Single-cell embedding graph separated by stages (E8.0, E8.25, E9.5 and E10.5), colored by clusters. Cluster colors are consistent with those in Fig. 2a.

b. Single-cell embedding graph separated by stages, colored by pseudotime. The color spectrum from red/orange is the early pseudotime point to blue/purple is late pseudotime point.

c. Single-cell embedding graph of the CPM lineages separated by stages, colored by clusters. Cluster colors are consistent with those in Fig. 2d.

d. Single-cell embedding graph colored by expression level. The color spectrum from blue, then green to yellow indicates expression levels from low to high. Gray indicates no expression. The genes are consistent with Fig. 2f.

e. Heatmap of average gene expression of the genes enriched in the cluster selected for the single-cell embedding graph. The genes are consistent with Fig. 2g.

f. Single-cell embedding graph colored by expression level of the marker genes of BrM, aSHF and pSHF in all clusters (top), CPM (middle) and MLPs (bottom). The color spectrum from blue, then green to yellow indicates expression levels from low to high. Gray indicates no expression.

Supplement figure 5

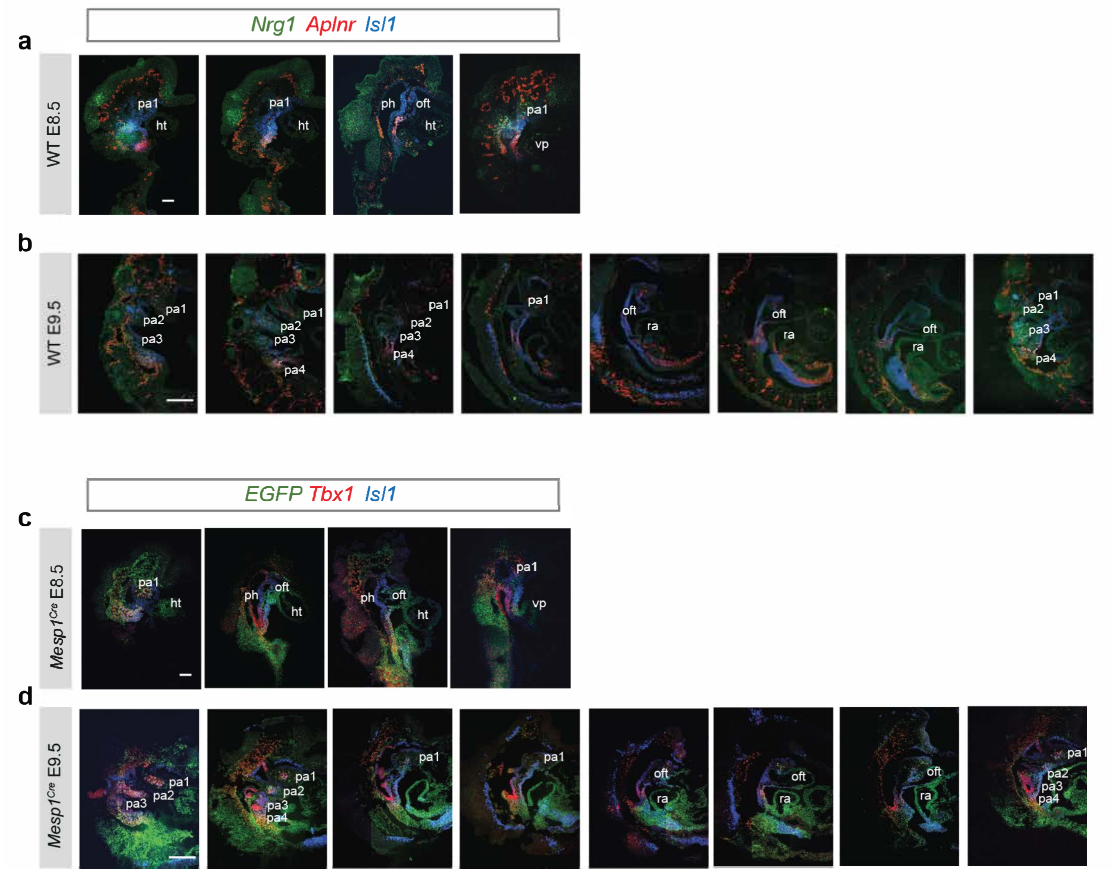

Supplementary Fig. 5. MLP marker gene expression in whole embryos

a. Serial RNAscope *in situ* hybridization pictures with *Nrg1*, *Aplnr* and *Isl1* mRNA probes on sagittal sections E8.5 (n = 3). All the sections are from same embryo as Fig. 2h. Green, *Nrg1*; Red, *Aplnr*; Blue, *Isl1*. Scale bar, 100 μm.

b. Serial RNAscope *in situ* hybridization pictures with *Nrg1*, *Aplnr* and *Isl1* mRNA probes on sagittal sections E9.5 (n = 3). All the sections are from same embryo as Fig. 2h. Green, *Nrg1*; Red, *Aplnr*; Blue, *Isl1*. Scale bar, 200 μm.

c. Serial RNAscope *in situ* hybridization with *EGFP*, marking *Mesp1*+ lineage cells, *Tbx1* and *Isl1* mRNA probes on sagittal sections from $Mesp1^{Cre}$;$ROSA26\text{-}GFP^{f/+}$ embryos at E8.5 (n = 3). All the sections are from same embryo as Fig. 2k. Green, *EGFP*; Red, *Tbx1*, Blue, *Isl1*. Scale bar, 100 μm.

d. Serial RNAscope *in situ* hybridization with *EGFP*, marking *Mesp1*+ lineage cells, *Tbx1* and *Isl1* mRNA probes on sagittal sections from $Mesp1^{Cre}$;$ROSA26\text{-}GFP^{f/+}$ embryos at E9.5 (n = 3). All the sections are from same embryo as Fig. 2k. Green, *EGFP*; Red, *Tbx1*, Blue, *Isl1*. Scale bar, 200 μm. Abbreviations: heart (ht), outflow tract (oft), pharyngeal arch (PA), pharynx (ph), right ventricle (RA), venous pole (vp), 1, 2 and 3 indicate the first, second and third pharyngeal arches, respectively.

Supplement figure 6

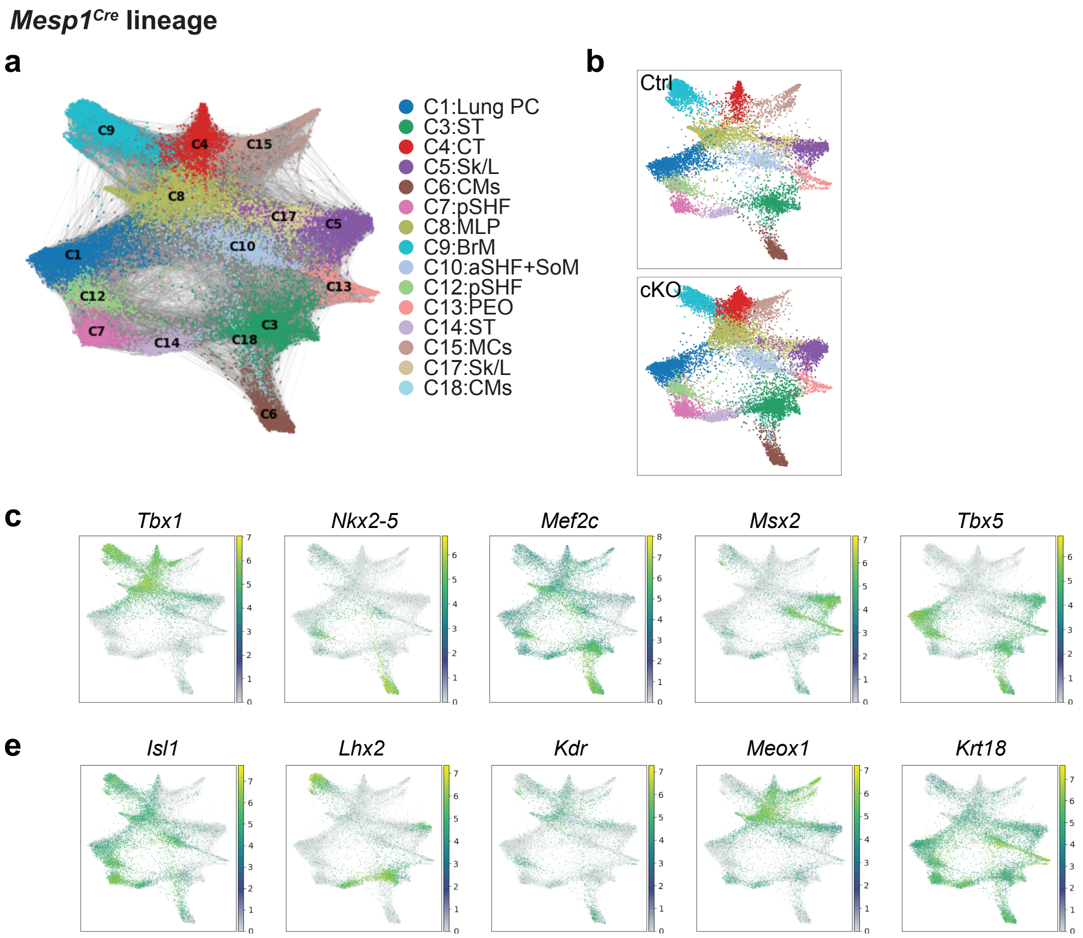

Supplementary Fig. 6. Cluster identification of *Mesp1^{Cre}* Ctrl and *Tbx1* cKO at E9.5 datasets

a. Single-cell embedding graph with PAGA plot of the integrated dataset from two replicates of *Mesp1$^{Cre/+}$;ROSA26-GFP$^{f/+}$* (Ctrl) vs *Mesp1$^{Cre/+}$;ROSA26-GFP$^{f/+}$;Tbx1$^{f/f}$* (cKO) embryos at E9.5, colored by cluster. C1: Lung PC, lung progenitors; C3: ST, septum transversum; C4: CT, connective tissues; C5: Sk/L, skeleton/limb; C6: CMs, cardiomyocyte progenitor cells; C7: pSHF, posterior CPM; C8: MLP, multilineage progenitors; C9: BrM, branchiomeric muscle progenitors; C10: aSHF/SoM, anterior CPM and somatic mesoderm; C12: pSHF, posterior CPM; C13: PEO, proepicardial organ; C14: ST, septum transversum; C15: MCs, mesenchyme cells; C17: Sk/L, skeleton/limb; C6: OFT, cardiomyocyte progenitor cells in OFT. CPM clusters are in bold font.

b. Single-cell embedding graph separated by genotype, colored by clusters.

c. Single-cell embedding graph colored by expression level. The color spectrum from blue to yellow indicates expression levels from low to high. Gray indicates no expression.

d. Single-cell embedding graph colored by expression level of the marker genes. The color spectrum from blue to yellow indicates expression levels from low to high. Gray indicates no expression.

Supplement figure 7

***Tbx1*^{Cre} lineage**

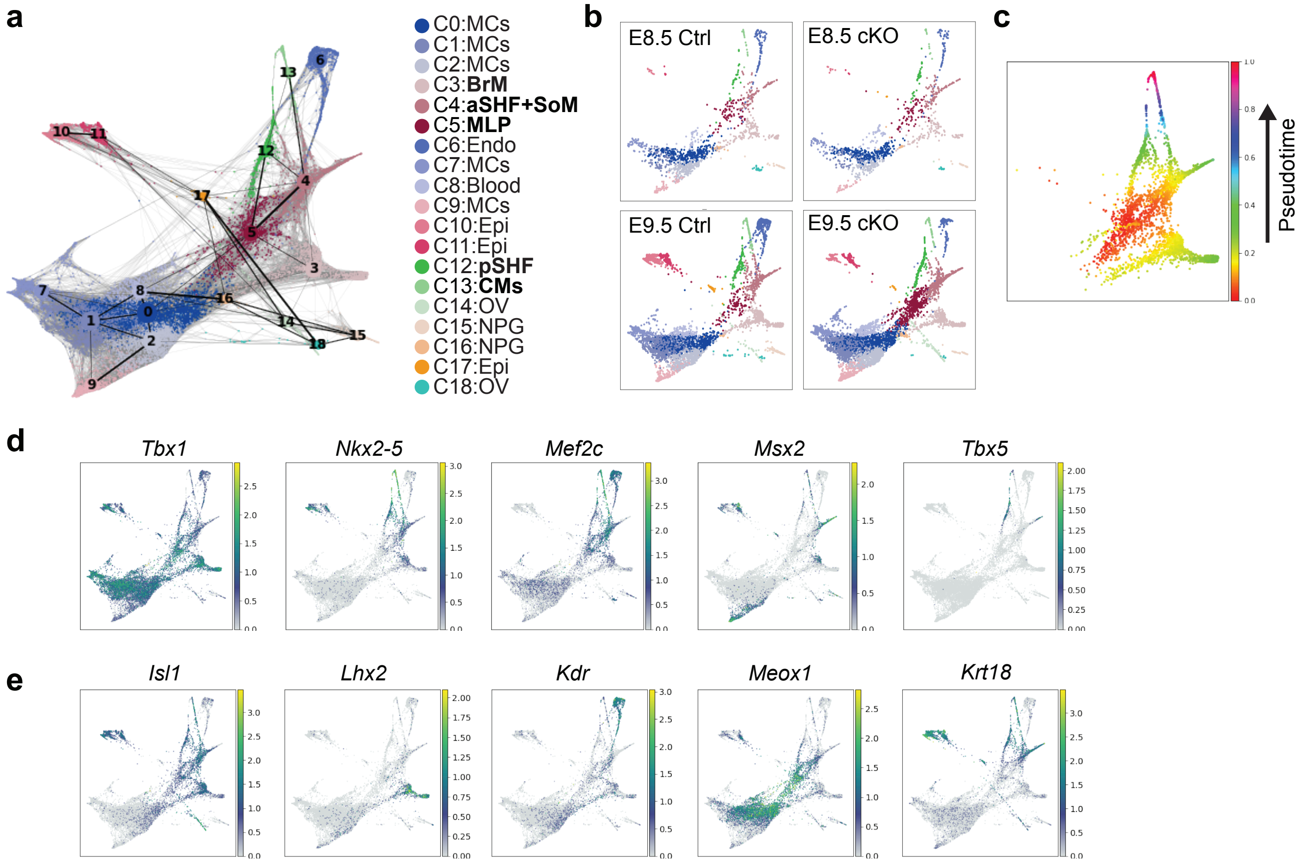

Supplementary Fig. 7. Cluster identification of *Tbx1*^{Cre} Ctrl and cKO embryos at E8.5 and E9.5.

a. Single-cell embedding graph with PAGA plot of the integrated dataset from *Tbx1*$^{Cre/+}$*;ROSA26-GFP*$^{f/+}$ (Ctrl) vs *Tbx1*$^{Cre/f}$*; ROSA26-GFP*$^{f/+}$ (cKO) embryos at E8.5 and E9.5, colored by cluster. C0: MCs, mesenchyme; C1: MCs, mesenchyme; C2: MCs, mesenchyme; C3: BrM, branchiomeric muscle progenitor cells; C4: aSHF/SoM, anterior CPM and somatic mesoderm; C5: MLP, multi-lineage progenitor cells; C6: Endo, endocardium and endothelial cells; C7: MCs, mesenchyme; C8: Blood, blood cells; C9: MCs, mesenchyme; C10: Epi, epithelia including endoderm and ectoderm; C11: Epi, epithelia including endoderm and ectoderm; C12: pSHF, posterior CPM; C13: CMs, cardiomyocyte progenitor cells; C14: OV, otic vesicle; C15: NPG, cochlear-vestibular ganglion neural progenitor cells; C15: NPG, cochlear-vestibular ganglion neural progenitor cells; C17: Epi, epithelia including endoderm and ectoderm; C18: OV, otic vesicle. CPM clusters are in bold font.

b. Single-cell embedding graph separated by genotype and stage, colored by clusters. The color spectrum from blue to yellow indicates expression levels from low to high. Gray indicates no expression.

c. Single-cell embedding graph of CPM clusters colored by pseudotime. The color spectrum from red/orange is early pseudotime point to blue/purple as late pseudotime point.

d and **e**. Single-cell embedding graph colored by expression level, related to Fig. 5h. The color spectrum from blue to yellow indicates expression level from low to high. Gray indicates no expression.

Supplement figure 8

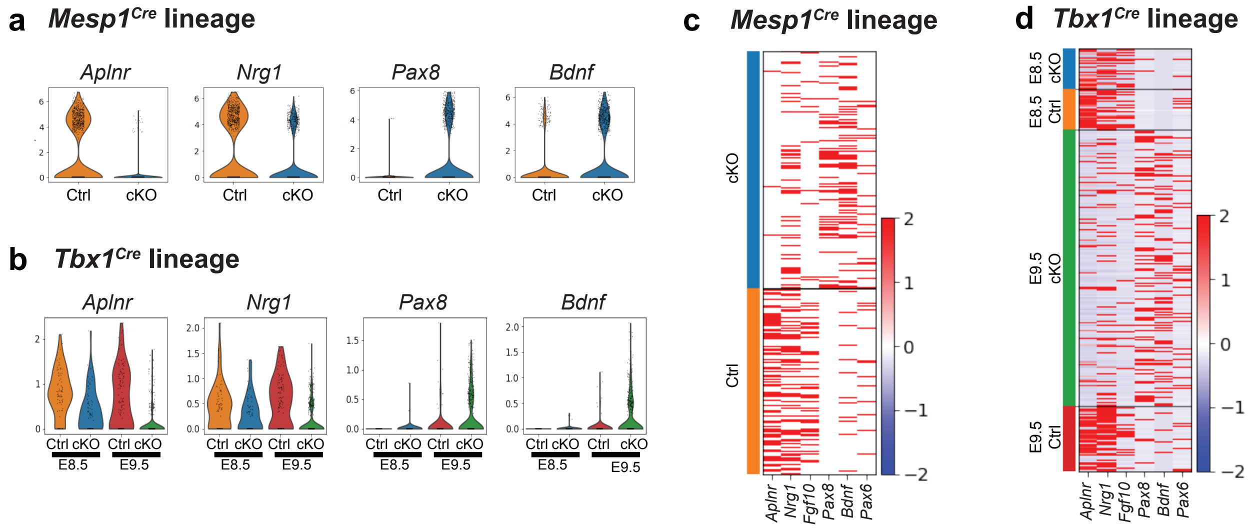

Supplementary Fig. 8. Gene expression of DEGs in CPM lineages

a. Violin plot of *Aplnr*, *Nrg1*, *Pax8* and *Bdnf* expression in MLPs from *Mesp1*^{Cre} Ctrl and cKO data at E9.5.

b. Violin plot of *Aplnr*, *Nrg1*, *Pax8* and *Bdnf* expression in MLP from *Tbx1*^{Cre} Ctrl and cKO at data at E8.5 and E9.5.

c. Heatmap of expression of *Aplnr*, *Nrg1*, *Pax8*, *Bdnf* and *Pax6* in MLPs from *Mesp1*^{Cre} Ctrl and cKO data at E9.5. Row indicates the expression of each cell.

d. Heatmap of expression of *Aplnr*, *Nrg1*, *Pax8*, *Bdnf* and *Pax6* in MLPs from *Tbx1*^{Cre} Ctrl and cKO data at E8.5 and E9.5. Row indicates the expression of each cell.

Supplement figure 9

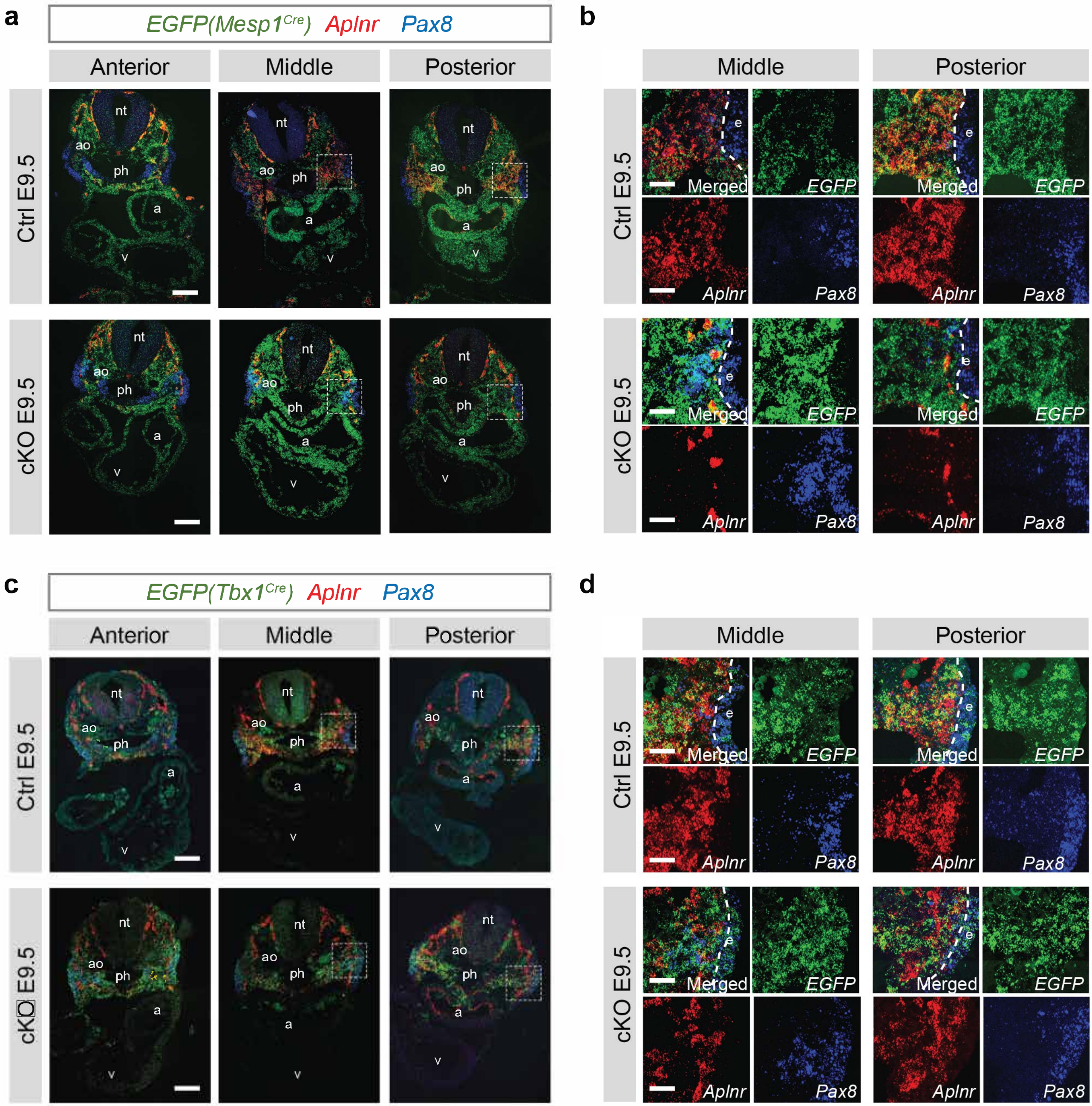

Supplementary Fig. 9. Gene expression patterns of *Aplnr* and *Pax8* at E9.5
a. RNAscope *in situ* hybridization with *EGFP*, *Aplnr* and *Pax8* mRNA probes on transverse section from *Mesp1*$^{Cre/+}$*;ROSA26-GFP*$^{f/+}$*;Tbx1*$^{f/+}$ (Ctrl) embryos (upper panel; n = 3) and *Mesp1*$^{Cre/+}$*;ROSA26-GFP*$^{f/+}$*;Tbx1*$^{f/f}$ (cKO) embryos (lower panel; n = 3) at E9.5. Green; *GFP*, Red; *Aplnr*, Blue; *Pax8*. Scale bar, 100 μm. White dotted line indicates the position in higher magnification images in **b**.
b. Higher magnification images from **a** (n = 3). Upper left, merged image; Upper right, green channel (*GFP*), Lower left, red channel (*Aplnr*); Lower right, blue channel (*Pax8*). Dotted line indicates the boundary of ectoderm and mesoderm. Scale bar, 30 μm.
c. RNAscope *in situ* hybridization with *GFP*, *Aplnr* and *Pax8* mRNA probes on transverse section from *Tbx1*$^{Cre/+}$*;ROSA26-GFP*$^{f/+}$ (Het, Ctrl) embryos (upper panel; n = 3) and *Tbx1*$^{Cre/f}$*;ROSA26-GFP*$^{f/+}$ (cKO) embryos (lower panel; n = 3) at E9.5. Green; *GFP*, Red; *Aplnr*, Blue; *Pax8*. Scale bar, 100 μm. White dotted line indicates the position in higher magnification images.
d. Higher magnification images of **c** (n = 3). Upper left, merged image; Upper right, green channel (*EGFP*), Lower left, red channel (*Aplnr*); Lower right, blue channel (*Pax8*). Scale bar; 30 μm. Dotted line indicates the boundary of ectoderm and mesoderm. Abbreviations: atrium (a), aorta (ao), ectoderm(e), neural tube (nt), pharynx (ph), ventricle (v).

Supplement figure 10

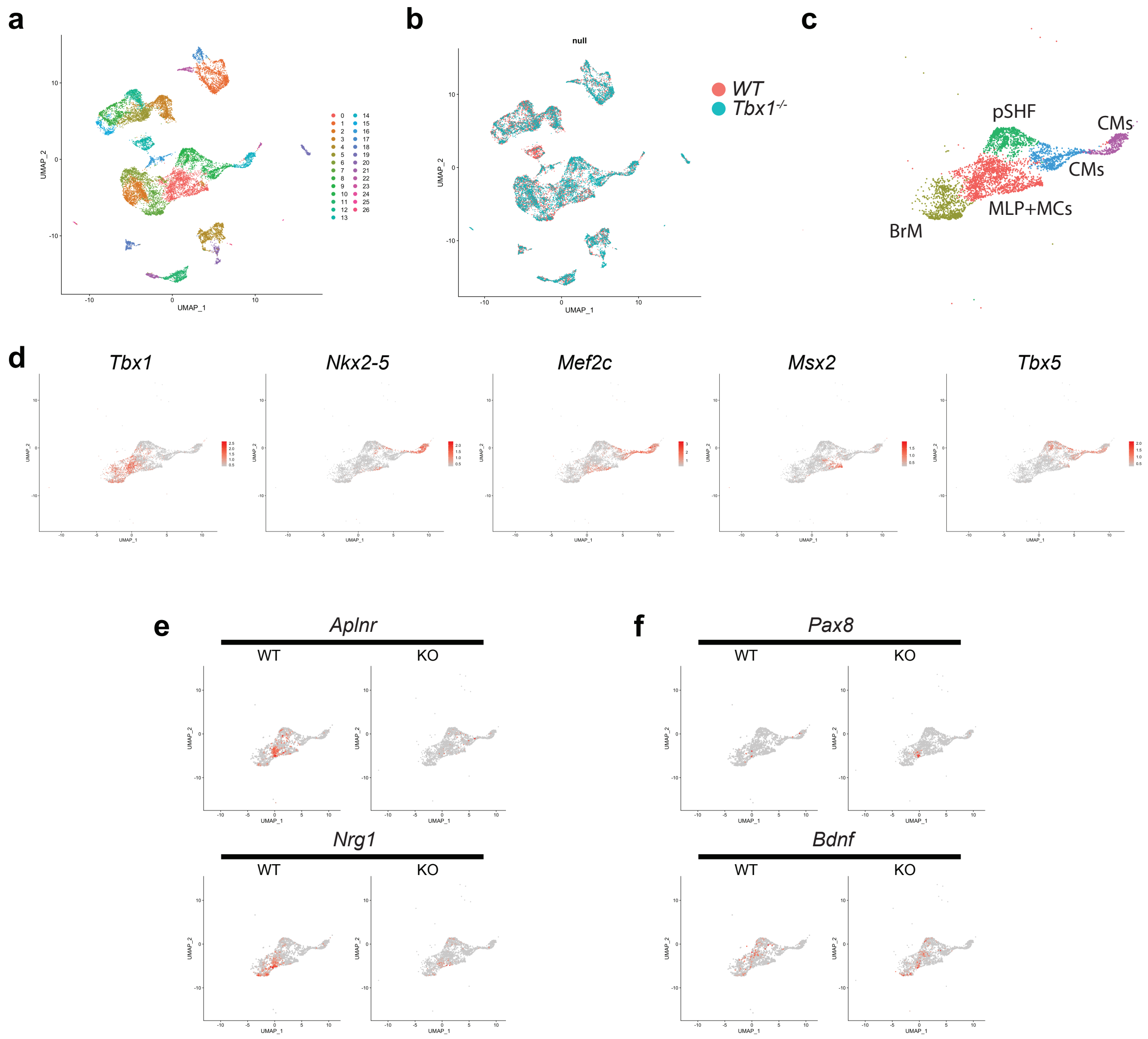

Supplementary Fig. 10. MLPs with similar gene expression changes occur in global *Tbx1* null mutant embryos at E9.5

a. UMAP plot of scRNA-seq data from SwissWebster wildtype (WT) versus *Tbx1*$^{-/-}$ embryos (KO) at E9.5. The pharyngeal apparatus plus heart were dissected, without lineage purification. Individual clusters are indicated by color.

b. UMAP plot colored by genotype (WT, coral; *Tbx1*$^{-/-}$, aqua).

c. UMAP plot of CPM population colored by cluster. The MLPs are mixed with mesenchymal cell types (MCs).

d. UMAP plots of CPM population colored by gene expression level. The color spectrum from gray to red indicates expression levels from low to high.

e. UMAP plots of CPM population colored by gene expression level of *Aplnr* and *Nrg1*, separated by genotype.

f. UMAP plots of CPM population colored by gene expression level of *Pax8* and *Bdnf*, separated by genotype.

Supplement figure 11

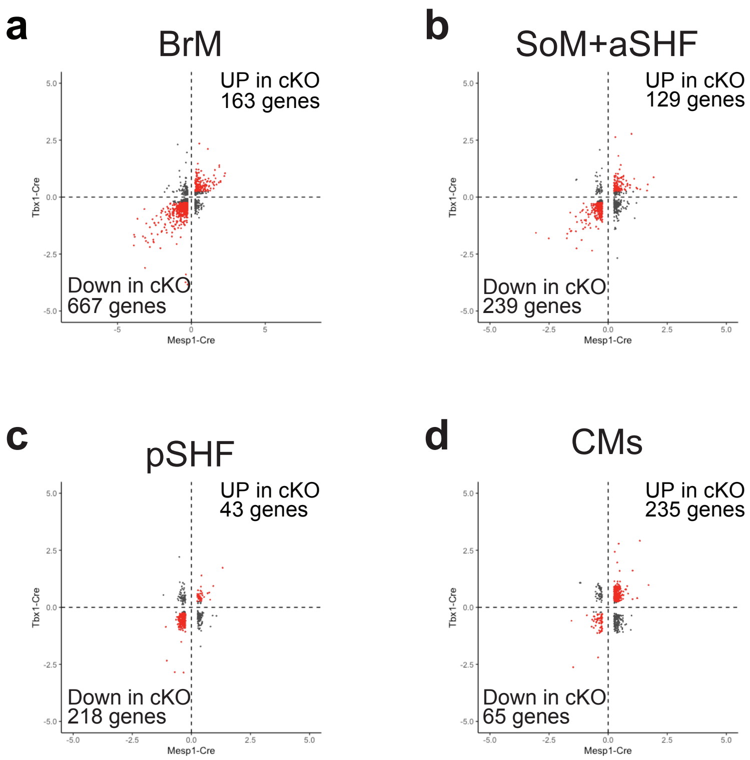

Supplementary Fig. 11. Differentially expressed genes analysis in CPM lineages
Comparison of differentially expressed genes (DEGs) from *Mesp1*$^{Cre/+}$*; ROSA26-GFP*$^{f/+}$ (Ctrl) vs *Mesp1*$^{Cre/+}$*; ROSA26-GFP*$^{f/+}$*;Tbx1*$^{f/f}$ (cKO) embryos at E9.5 and *Tbx1*$^{Cre/+}$*; ROSA26-GFP*$^{f/+}$ (Ctrl) vs *Tbx1*$^{Cre/f}$*; ROSA26-GFP*$^{f/+}$ (cKO) in BrM (**a**), aSHF/SoM (**b**), pSHF(**c**) and CMs (**d**). X-axis indicates log2-fold change of *Mesp1*^{Cre} DEGs. Y-axis indicates log2-fold change of *Tbx1*^{Cre} DEGs. Each dot indicates a gene, and red indicates common DEGs with |log2-fold change| > 0.25 in both comparisons. Cluster, C12 in the *Mesp1*^{Cre} dataset and cluster, C12 in the *Tbx1*^{Cre} dataset were used for pSHF DEG analysis.

Supplement figure 12

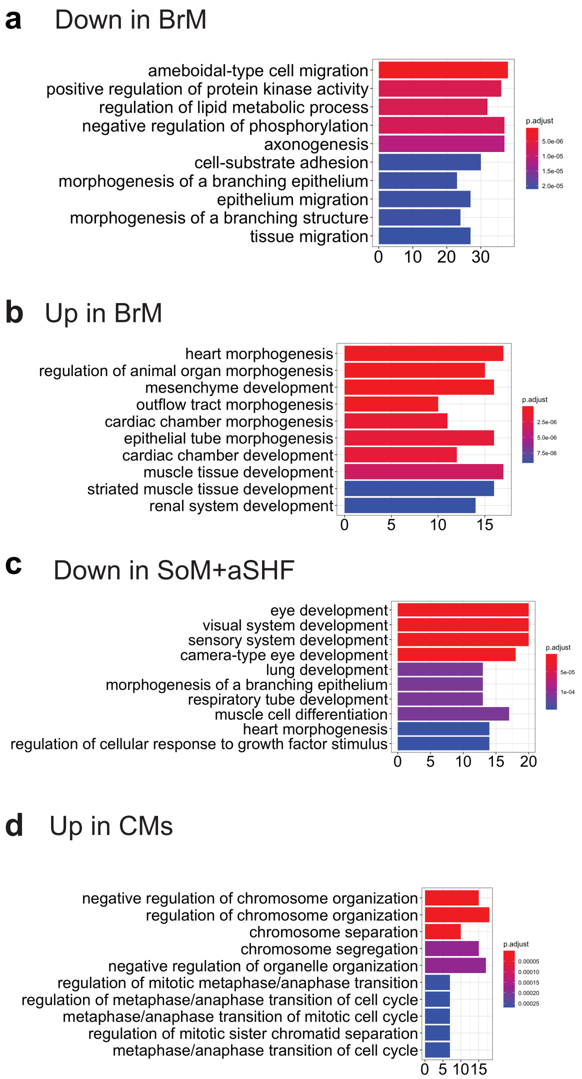

Supplementary Fig. 12. Gene Ontology analysis of differentially expressed genes analysis in CPM lineages

a and **b.** Enriched biological processes based upon GO found in downregulated (**a**) and upregulated (**b**) genes in BrM cells. X-axis indicates the number of genes in the pathway. The color of bar chart indicates adjusted p-value.

c. Enriched biological processes based upon GO found in downregulated genes in the aSHF/SoM cluster. X-axis indicates the number of genes in the pathway. The color of bar chart indicates adjusted p-value.

d. Enriched biological processes based upon GO found in upregulated genes in the CM cluster. X-axis indicates the number of genes in the pathway. The color of bar chart indicates adjusted p-value.

Note: There was no enriched GO found in upregulated genes in aSHF/SoM, both in pSHF and downregulated genes in CMs of *Tbx1* cKO data.

Supplement figure 13

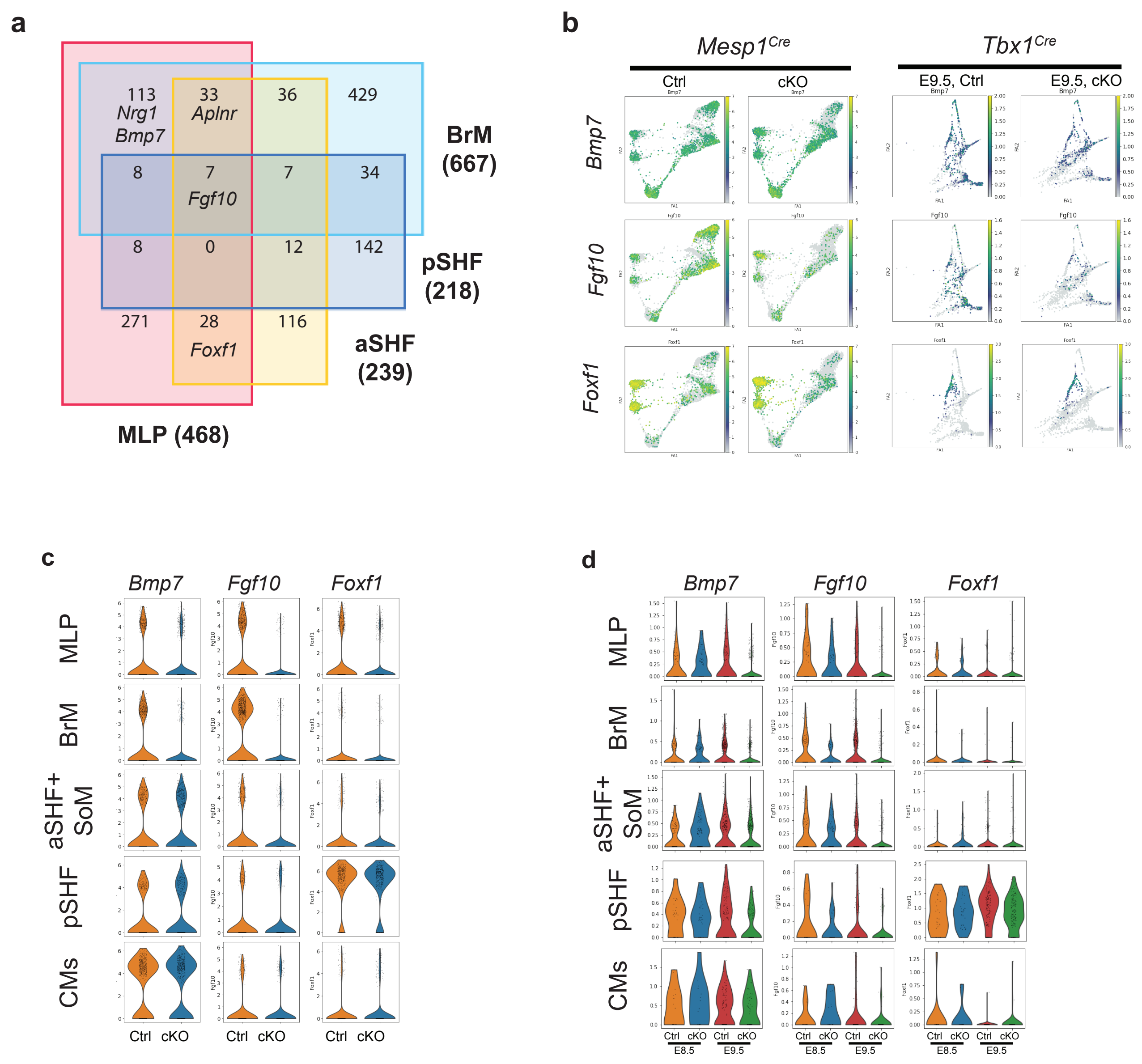

Supplementary Fig. 13. Comparison of DEGs in CPM derivatives with MLPs
a. Venn diagram of downregulated genes in CPM clusters of *Mesp1*^{Cre}; Ctrl vs cKO and *Tbx1*^{Cre} Ctrl vs *Tbx1* cKO embryos. The downregulated genes in MLPs, BrM, aSHF and pSHF populations were compared. The number in the diagram indicates the number of genes in each category. The full list is available in Supplemental Tables 7 and 9.
b. Single-cell embedding graph of the CPM lineages from *Mesp1*^{Cre} Ctrl vs *Tbx1* cKO at E9.5 datasets and *Tbx1*^{Cre} Ctrl vs cKO datasets at E9.5 separated by genotype and colored by expression level. The color spectrum from blue to yellow indicates expression levels from low to high. Gray indicates no expression.
c. Violin plot of *Bmp7*, *Fgf10* and *Foxf1* in each CPM cluster from *Mesp1*^{Cre} Ctrl vs cKO data at E9.5.
d. Violin plot of *Bmp7*, *Fgf10* and *Foxf1* in each CPM cluster from *Tbx1*^{Cre} Ctrl and cKO data at E8.5 and E9.5.

Supplement figure 14

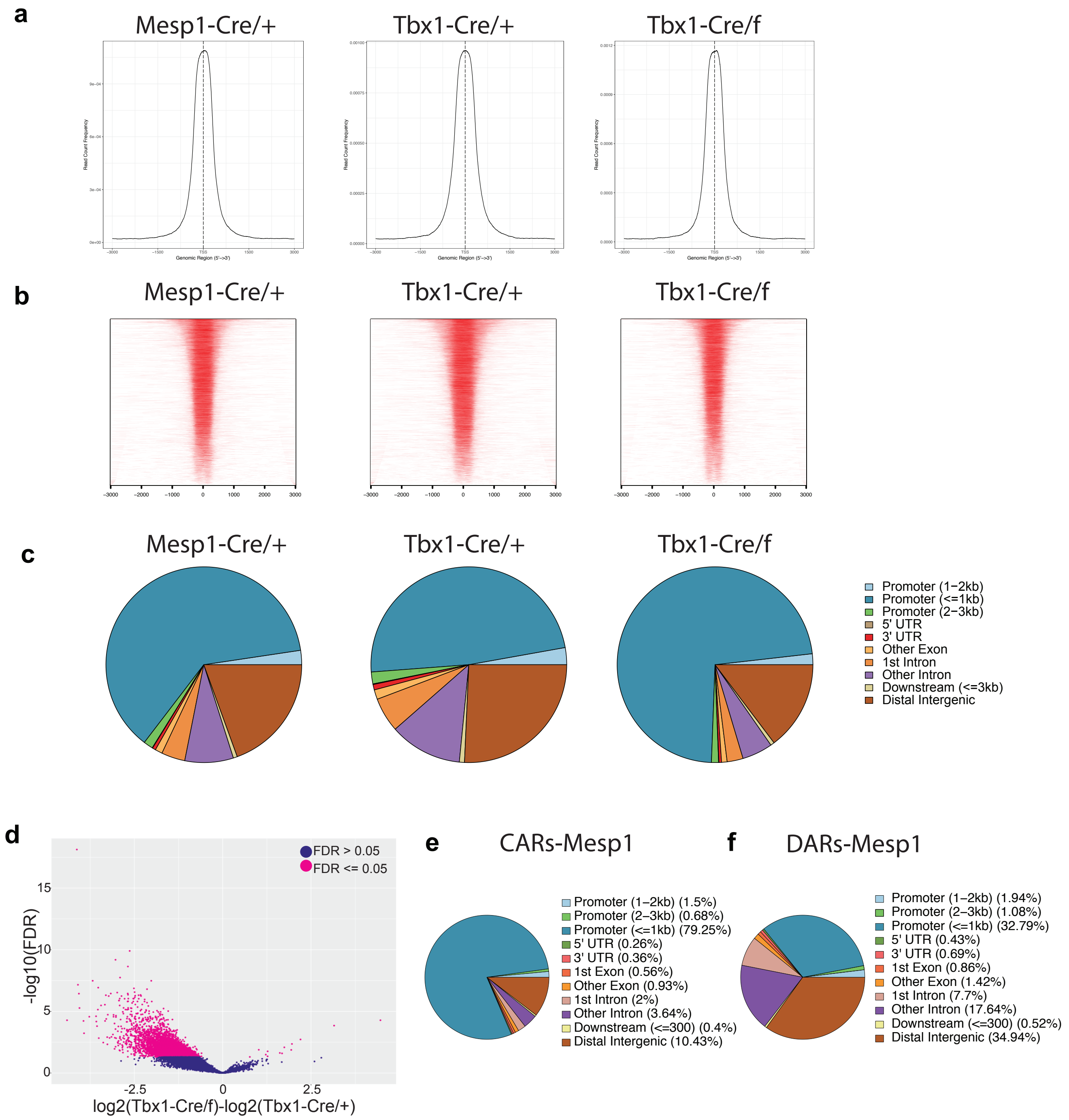

Supplementary Fig. 14. ATAC-seq data analysis

a. Average ATAC-seq read densities at regions ±3kb from transcription start sites of GFP+ cells from *Mesp1*$^{Cre/+}$*; ROSA26-GFP*$^{f/+}$ (Left), *Tbx1*$^{Cre/+}$*; ROSA26-GFP*$^{f/+}$ (Middle) and *Tbx1*$^{Cre/f}$*; ROSA26-GFP*$^{f/+}$ (Right).

b. Heatmap of ATAC-seq read densities at regions ±3kb from transcription start sites of GFP+ cells from *Mesp1*$^{Cre/+}$*; ROSA26-GFP*$^{f/+}$ (Left), *Tbx1*$^{Cre/+}$*; ROSA26-GFP*$^{f/+}$ (Middle) and *Tbx1*$^{Cre/f}$*; ROSA26-GFP*$^{f/+}$ (Right).

c. Pie chart of genome distribution of ATAC-seq peaks of GFP+ cells from *Mesp1*$^{Cre/+}$*; ROSA26-GFP*$^{f/+}$ (Left), *Tbx1*$^{Cre/+}$*; ROSA26-GFP*$^{f/+}$ (Middle) and *Tbx1*$^{Cre/f}$*; ROSA26-GFP*$^{f/+}$ (Right).

d. Volcano plot of the ATAC-seq peaks from *Tbx1*^{Cre} Ctrl vs *Tbx1*^{Cre} cKO dataset. The pink dots indicate differentially accessible regions (FDR ≤ 0.05)

e. Pie chart of genome distribution of CAR-Mesp1.

f. Pie chart of genome distribution of DAR-Mesp1.

Supplement figure 15

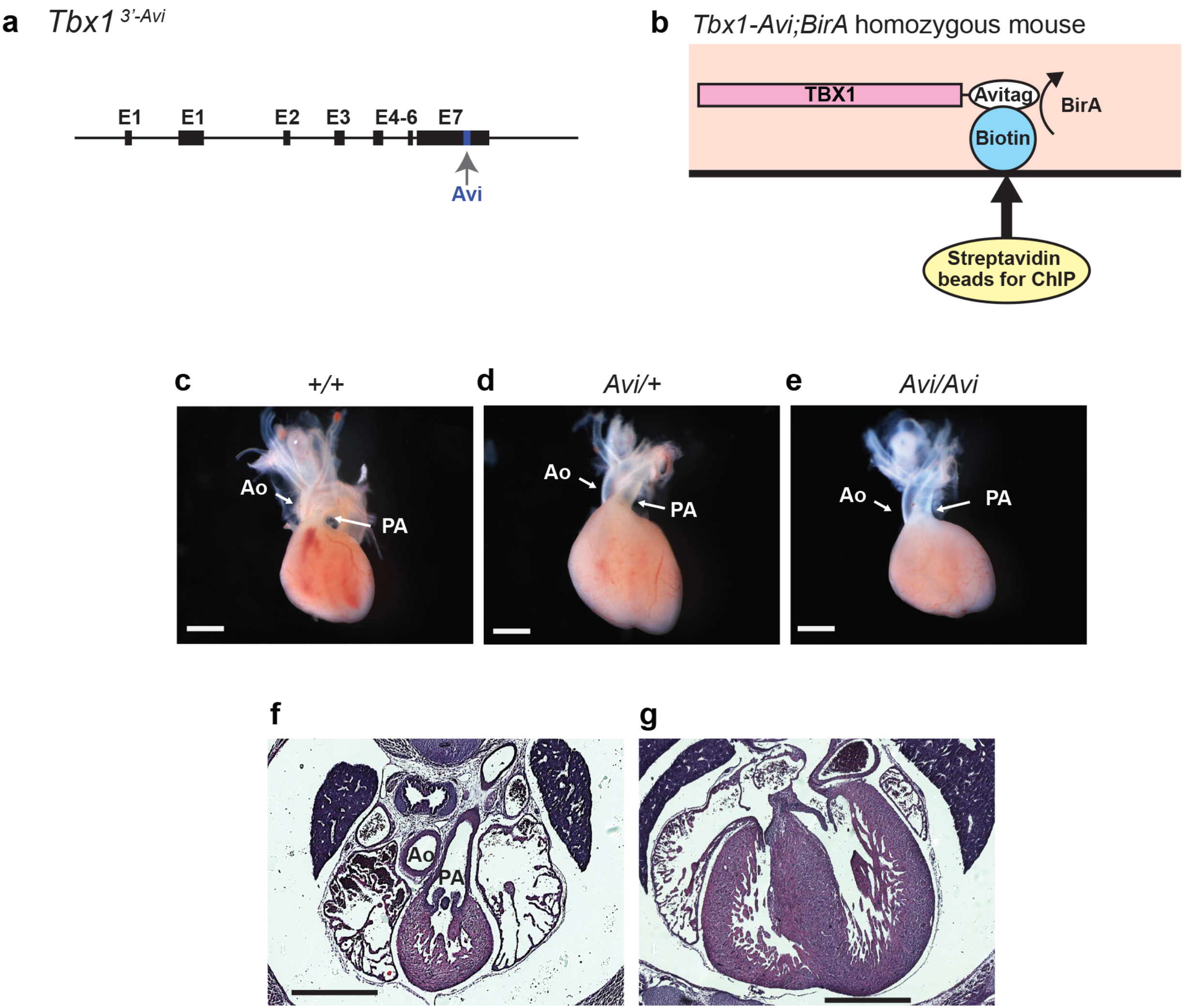

Supplementary Fig. 15. *Tbx1-Avi* mouse for ChIP-seq

a. *Tbx1* gene modification for *Tbx1-Avi* mouse**.** The Avi-tag sequence was inserted in 3' end of *Tbx1* exon 7.

b. Scheme of TBX1 *in vivo* ChIP-seq with Avi-tag. In the $Tbx1^{Avi/Avi}$*;BirA/BirA* homozygous mouse, biotin was bind to Avi-tag of the TBX1 protein.

c-**e.** Wildtype, $Tbx1^{Avi/+}$ and $Tbx1^{Avi/Avi}$ littermates have normal hearts - aorta (Ao), pulmonary artery (PA) at E16.5. Scale bar, 1 mm.

f and **g.** H&E staining of $Tbx1^{Avi/Avi}$ transverse sections of hearts at E16.5 (n = 3; Scale bar, 1 mm).

Supplement figure 16

a

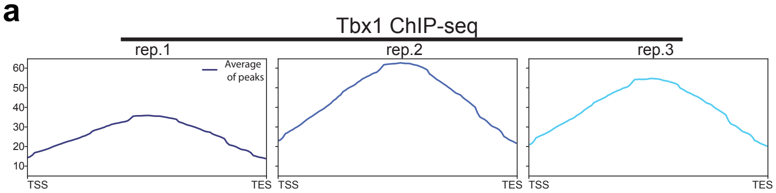

b

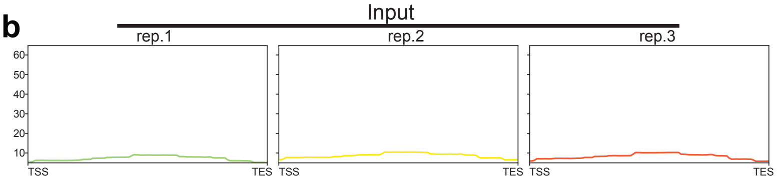

c

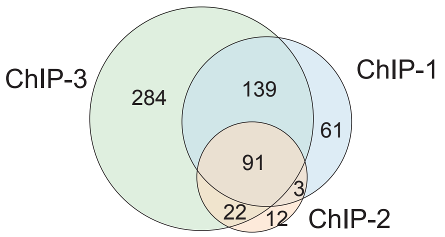

d known

Rank	Motif	Transcription factor	p-value	Back ground	Targets
1	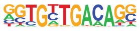	Tbx20(T-box)	1e-59	4.44%	37.25%
2	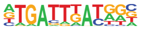	PBX2(Homeobox)	1e-36	13.08%	45.88%
3	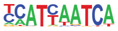	Pdx1(Homeobox)	1e-34	16.16%	49.80%
4	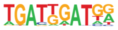	HOXA1(Homeobox)	1e-33	5.47%	29.41%

e *De novo*

Rank	Motif	Transcription factor	p-value	Back ground	Targets
1	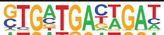	Tbx20(T-box)	1e-134	3.71%	56.86%
2	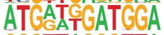	HOXA1(Homeobox)	1e-25	0.12%	7.06%
3	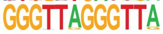	PSE(SNAPc)	1e-12	0.01%	2.35%

f

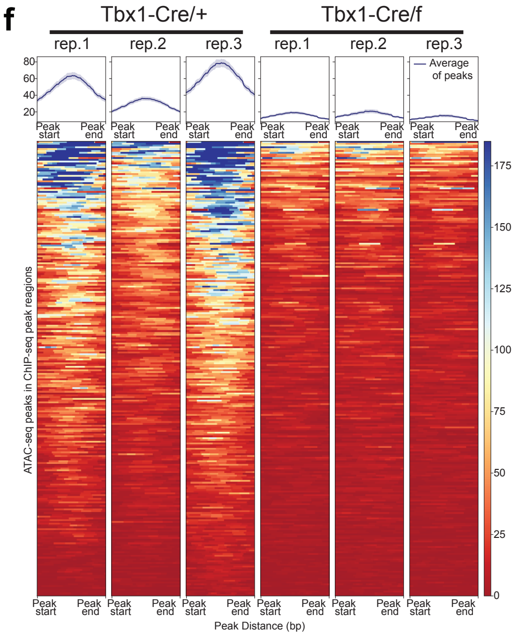

Supplementary Fig. 16. ChIP-seq data analysis
a. Average Tbx1-ChIP read densities of each replicate at regions from transcription start sites (TSS) to transcription end site (TES).
b. Average input read densities of each replicate at regions from transcription start sites (TSS) to transcription end site (TES).
c. Venn diagram of peaks from three replicates of ChIP-seq. We used the peaks found in at least two replicates.
d. The known motifs found in ChIP-seq regions by Homer software.
e. The novel motifs found in ChIP-seq regions by Homer software.
f. Chromatin accessibility of ChIP-seq peak regions in three replicates of ATAC-seq data. Left, ATAC-seq data from *Tbx1*^{Cre} Ctrl, Right, ATAC-seq data from *Tbx1*^{Cre} cKO data.
