# Supplementary material for: Single cell multi-omic analysis identifies a Tbx1-dependent multilineage primed population in murine cardiopharyngeal mesoderm
Source: Nat Commun. 2021 Nov 17;12:6645. doi: 10.1038/s41467-021-26966-6 (PMC8599455; doi:10.1038/s41467-021-26966-6)
Supplement: Supplementary file 3 — Description of Additional Supplementary Files [file 41467_2021_26966_MOESM3_ESM.pdf]

Supplementary Data 1. Marker genes of cell clusters *in Mesp1^{Cre}* four timepoint dataset. Gene expression data used to identify the individual cell clusters among the 20 as shown in Fig. 1.

Supplementary Data 2. Marker genes of cell clusters *in Mesp1^{Cre}* Ctrl and *Tbx1^{Cre}* Ctrl at E9.5. Gene expression data used to identify cell clusters in integrated data from *Mesp1^{Cre}* and *Tbx1^{Cre}* Ctrl embryos at E9.5 as related to data shown in Fig. 4.

Supplementary Data 3. Marker genes of cell clusters *in Mesp1^{Cre}* Ctrl and cKO at E9.5. Gene expression data used to identify cell clusters in the integrated data from *Mesp1^{Cre}* Ctrl and cKO embryos at E9.5 as related to Fig. 5c-d, g.

Supplementary Data 4. Differentially expressed genes (DEGs; adjusted p-value < 0.05) in each cluster of *Mesp1^{Cre}* Ctrl and *Mesp1^{Cre}* cKO at E9.5. DEGs identified in *Mesp1^{Cre}* Ctrl and *Mesp1^{Cre}* cKO embryos used to generate Figs. 5-7.

Supplementary Data 5. Marker genes of cell clusters in *Tbx1^{Cre}* Ctrl and cKO embryos at E8.5 and E9.5. Marker genes identified in integrated data from *Tbx1^{Cre}* Ctrl and *Tbx1^{Cre}* cKO embryos used to generate Fig. 5e, f, h.

Supplementary Data 6. Differentially expressed genes (DEGs; adjusted p-value < 0.05) in each cluster of *Tbx1^{Cre}* Ctrl and cKO at E9.5. DEGs identified in *Tbx1^{Cre}* Ctrl and *Tbx1^{Cre}* cKO embryos used to generate Figs. 5-8.

Supplementary Data 7. Differentially expressed genes (DEGs; adjusted p-value < 0.05, fold change > |0.25| in both *Mesp1^{Cre}* Ctrl vs cKO at E9.5 data and *Tbx1^{Cre}* Ctrl vs cKO at E9.5 data) in MLPs. Data from two replicates of *Mesp1^{Cre}* Ctrl vs cKO and one *Tbx1^{Cre}* Ctrl vs cKO experiment were integrated and DEGs were identified between both Ctrl vs cKO mutant embryos. Study design is shown in Fig. 6a; data was used to generate Figs. 6-8.

Supplementary Data 8. Gene ontology biological pathway found in DEGs of MLPs. Gene ontology pathways identified from DEGs that were decreased or increased in expression in cKO embryos obtained from Supplementary Table 7. The results are shown in Figs. 6-8.

Supplementary Data 9. Differentially expressed genes (DEGs; adjusted p-value < 0.05, fold change > |0.25| in both *Mesp1^{Cre}* Ctrl vs cKO at E9.5 data and *Tbx1^{Cre}* Ctrl vs cKO at E9.5 data) in BrM, aSHF/SoM, pSHF and CMs. DEGs were identified in other CPM cell populations, excluding MLPs (Supplementary Data 7).

Supplementary Data 10. Gene ontology biological pathway found in DEGs of CPM cells (BrM, aSHF/SoM and CMs). Gene ontology pathways identified from DEGs that were decreased or increased in expression in cKO embryos obtained from Supplementary Table 9. The results are shown in Supplementary Figs. 11-13.

Supplementary Data 11. The motif enriched in DARs-Mesp1 ATAC-seq peak regions. Transcription factor binding motifs that were enriched in DARs-Mesp1 peaks identified in the ATAC-seq dataset.

Supplementary Data 12. DARs-Mesp1 ATAC-seq peak regions with annotated genes. DARs-Mesp1 from the ATAC-seq dataset around gene loci. DEGs *Tbx1* (column G) and *Mesp1* (column H) with direction (column I) are also added for comparison.

Supplementary Data 13. The motif enriched in TBX1 ChIP-seq peak regions. Transcription factor DNA binding motifs that are co-localized with TBX1 ChIP-seq regions.

Supplementary Data 14. TBX1 ChIP-seq peak regions with annotated genes. Genes that contain TBX1 transcription factor binding sites identified from TBX1 ChIP-seq. Chromosome peak start/end is listed (columns B-D). Peaks that are intergenic are linked to more than one gene, explaining the difference in number of genes vs peaks. Distance from TSS is indicated (column F). The 21 genes from the intersection of DEGs/DARs/ChIP peaks, shown in Fig. 8b are listed (column G).
