# Supplementary material for: Single cell multi-omic analysis identifies a Tbx1-dependent multilineage primed population in murine cardiopharyngeal mesoderm
Source: Nat Commun. 2021 Nov 17;12:6645. doi: 10.1038/s41467-021-26966-6 (PMC8599455; doi:10.1038/s41467-021-26966-6)
Supplement: Supplementary file 18 — Reporting Summary [file 41467_2021_26966_MOESM18_ESM.pdf]

nature portfolio

Corresponding author(s): Bernice Morrow

Last updated by author(s): Oct 14, 2021

Reporting Summary

Nature Portfolio wishes to improve the reproducibility of the work that we publish. This form provides structure for consistency and transparency in reporting. For further information on Nature Portfolio policies, see our Editorial Policies and the Editorial Policy Checklist.

Statistics

For all statistical analyses, confirm that the following items are present in the figure legend, table legend, main text, or Methods section.

n/a	Confirmed	
☐	☒	The exact sample size (n) for each experimental group/condition, given as a discrete number and unit of measurement
☐	☒	A statement on whether measurements were taken from distinct samples or whether the same sample was measured repeatedly
☐	☒	The statistical test(s) used AND whether they are one- or two-sided<br>*Only common tests should be described solely by name; describe more complex techniques in the Methods section.*
☐	☒	A description of all covariates tested
☐	☒	A description of any assumptions or corrections, such as tests of normality and adjustment for multiple comparisons
☐	☒	A full description of the statistical parameters including central tendency (e.g. means) or other basic estimates (e.g. regression coefficient) AND variation (e.g. standard deviation) or associated estimates of uncertainty (e.g. confidence intervals)
☐	☒	For null hypothesis testing, the test statistic (e.g. F, t, r) with confidence intervals, effect sizes, degrees of freedom and P value noted<br>*Give P values as exact values whenever suitable.*
☒	☐	For Bayesian analysis, information on the choice of priors and Markov chain Monte Carlo settings
☐	☒	For hierarchical and complex designs, identification of the appropriate level for tests and full reporting of outcomes
☒	☐	Estimates of effect sizes (e.g. Cohen's d, Pearson's r), indicating how they were calculated

Our web collection on statistics for biologists contains articles on many of the points above.

Software and code

Policy information about availability of computer code

Data collection	RNAscope<br>Whole mount GFP images were captured on a SteREO Discovery.V12 microscope (Zeiss Corp). Images from sections were captured using Zeiss Axio Observer microscope with ApoTome (Zeiss Corp); Images from whole mounts were captured on a confocal Leica SP5 microscope (Leica Corp).<br><br>FACS:<br>A BD FACSAria II system (BD Bioscience) was used for flow cytometry.<br><br>Next generation sequencing<br>Sequencing data were collected on a Illumina HiSeq 2500, HiSeq 4000 and NovaSeq 6000.
Data analysis	Data analysis:<br>FACS: BD FACSDiva 8.0.1 software was used for flow cytometer data acquisition and analysis (https://www.bdbiosciences.com/en-us/products/software/instrument-software/bd-facsdiva-software)<br><br>Data analysis:<br>scRNA-seq:<br>Cell Ranger v 3.1.0 is an analysis pipeline used to process single-cell data (10x Genomics; https://support.10xgenomics.com),<br>densityClust v0.3 was used for density peak clustering (Rodriguez and Laio, PMID: 24970081; https://packages.debian.org/sid/riscv64/gnu-r/r-cran-densityclust),<br>Scran v1.10.2 was used to normalize scRNA-seq data (Lun, McCarthy and Marioni, PMID: 27900975; PMC5112579; https://bioconductor.org/

packages/release/bioc/html/scran.html),
R version 3.5.2 was used for data analysis (https://www.rstudio.com; https://www.r-project.org),
Seurat 3.1.5 was used to visualize cell cluters(Butler et al., PMID: 29608179, PMC6700744),
Scanpy v1.4.3 was used to visualize scRNA-seq data (Wolf, Angerer and Theis, PMID: 29409532, PMC5802054; https://github.com/theislab/scanpy),
clusterProfiler v3.10.1 was used for functional enrichment analysis and to visualize differentially expressed genes (Yu et al., 22455463; PMC3339379; https://guangchuangyu.github.io/software/clusterProfiler),
RISC software was used for data integration (Liu et al., PMID: 33767393; PMC8456427).

Data analysis:
ATAC-seq:
Bowtie2 2.3.4.3 was used to align sequencing reads to reference genomic sequences (Langmead and Salzberg, PMID: 22388286, PMC332281; http://guix.gnu.org/packages/bowtie-2.3.4.3/),
MACS2 2.1.2.1 with default parameters was used to identify transcription factor binding sites (Feng et al., PMID: 22936215; PMC3868217, https://pypi.org/project/MACS2/2.1.1.20160226/),
DiffBind 2.14.0 was used to determine differential binding sites between multiple ChIP-seq experiments (Ross-Innes et al., PMID: 22217937, PMC3272464, https://bioconductor.org/packages/release/bioc/html/DiffBind.html),
BEDTools 2.29 was used to examine genomic intervals from different files (Quinlan and Hall, PMID: 20110278; PMC2832824, https://bedtools.readthedocs.io/en/latest/content/installation.html),
Homer v4.10.3 was used for DNA binding site motif discovery (Heinz et al., PMID: 20513432; PMC28988526, http://homer.ucsd.edu/homer/),
IGV 2.4.8 was used to visualize data (Robison et al., PMID: 21221095; PMC3346182,.https://software.broadinstitute.org/software/igv/igvtools),
clusterProfiler v3.10.1 was used for gene annotation (Yu et al., 22455463; PMC3339379; https://guangchuangyu.github.io/software/clusterProfiler),
ngs.plot was used to visualize sequencing data (Shen et al., PMID: 24735413; PMC4028082, https://code.google.com/p/ngsplot/),
deepTools2 was used for analysis of sequencing data (PMID: 27079975; PMC4987876, https://deeptools.readthedocs.io/en/develop/),
ChIPpeakAnno v3.22.4 (Zhu et al., PMIC: 20459804; PMC30988059; http://bioconductor.org/packages/release/bioc/html/ChIPpeakAnno.html)
intersectBed was used to intersect overlaps of two files (Quinlan and Hall, PMID: 20110278; PMC2832824, https://bedtools.readthedocs.io/en/latest/content/installation.html; https://cran.r-project.org/web/packages/bedr/vignettes/Using-bedr.html)
GREAT was used to annotate non-coding regions (McLean et al., 2043; PMC4840234; http://great.stanford.edu/public/html/).

Data analysis:
ChIP-seq:
Cutadapt removes adapter, primers, poly-A tails and it was used to remove unwanted sequence (Martin, https://doi.org/10.14806/ej.17.1.200; https://cutadapt.readthedocs.io/en/stable/)
FASTQC (https://www.bioinformatics.babraham.ac.uk/projects/fastqc/),
Bowtie2 2.3.4.3 was used to align sequencing reads to reference genomic sequences (Langmead and Salzberg, PMID: 22388286, PMC332281; http://guix.gnu.org/packages/bowtie-2.3.4.3/),
MACS2 2.1.2.1 with default parameters was used to identify transcription factor binding sites (Feng et al., PMID: 22936215; PMC3868217, https://pypi.org/project/MACS2/2.1.1.20160226/),
BEDTools 2.29 was used to examine genomic intervals from different files (Quinlan and Hall, PMID: 20110278; PMC2832824, https://bedtools.readthedocs.io/en/latest/content/installation.html),
dplyr package in R was for data analysis (https://www.rdocumentation.org/packages/dplyr/versions/0.7.8),
IGV 2.4.8, was used to visualize data (https://software.broadinstitute.org/software/igv/igvtools),
ngs.plot was used to visualize sequencing data (Shen et al., PMID: 24735413; PMC4028082, https://code.google.com/p/ngsplot/),
deepTools2 was used for analysis of sequencing data (Ramirez, PMID: 27079975; PMC4987876, https://deeptools.readthedocs.io/en/develop/)

Custom codes:
We removed 3 places in the text where we stated we used custom R-scripts because we actually used standard methods. Line 722, we compared gene lists using excel. Lines 732 and 763, we did not use a customized R-script, but used software described in that section of the Methods. On line 774, we changed “standard R-scripts” to state that we used the dplyr package in R”. Since we did not develop custom computer codes, we do not have a “Code Availability” section.

For manuscripts utilizing custom algorithms or software that are central to the research but not yet described in published literature, software must be made available to editors and reviewers. We strongly encourage code deposition in a community repository (e.g. GitHub). See the Nature Portfolio guidelines for submitting code & software for further information.

Data

Policy information about availability of data

All manuscripts must include a data availability statement. This statement should provide the following information, where applicable:

- Accession codes, unique identifiers, or web links for publicly available datasets
- A description of any restrictions on data availability
- For clinical datasets or third party data, please ensure that the statement adheres to our policy

Files GSE167493, GSE16749,1 GSE173700, GSE173521 are now publicly released (as of October 10, 2021). All analyses are shown in the Supplemental Tables.

Field-specific reporting

Please select the one below that is the best fit for your research. If you are not sure, read the appropriate sections before making your selection.

☒ Life sciences ☐ Behavioural & social sciences ☐ Ecological, evolutionary & environmental sciences

For a reference copy of the document with all sections, see nature.com/documents/nr-reporting-summary-flat.pdf

Life sciences study design

All studies must disclose on these points even when the disclosure is negative.

Sample size	No statistical methods were used to determine sample size. Sample sizes were estimated based upon requirements for required optimal cell numbers for scRNA-seq, ATAC-seq and ChIP-seq. Sample sizes for scRNAseq were provided in Table 1 of the manuscript, in which we used multiple time points and two different mouse lines to validate our findings. All In situ hybridization studies were done in n>3 as standard. ATAC-seq was done in 2 replicates and ChIP-seq was done in three replicates. For each replicate, we included 2 or more embryos per pool of the same genotype.
Data exclusions	Exclusion criteria were not pre-established. All data was included from the initial analyses and they are provided in GEO and Supplementary Data. For secondary analyses, we focused upon the cardiopharyngeal mesoderm (CPM) cell populations. Data that was not shared between the 2 different Cre lines used was excluded for secondary analysis because they are not CPM cells.
Replication	Our replication attempts were successful. All experimental findings were reproduced independently 2 or 3 times. We used 2 different Cre lines for our experiments as a replication of each other. Also, we used another scRNA-seq experiment of global null versus wildtype embryos.
Randomization	Mice were randomly allocated into experimental groups based upon genotype. Female and male mice for genetic crosses were sorted randomly, at 2-6 months of age, during peak of reproduction. Mouse embryos were genotyped to determine whether they were mutant vs wildtype. Further, we examined the embryos to make sure that genotype matched phenotype.
Blinding	We genotyped mice and embryos to know their affection status and this was needed to do functional genomic studies. scRNA-seq, ChIP-seq and ATAC-seq uses software that provides unbiased clustering and peaks.

Reporting for specific materials, systems and methods

We require information from authors about some types of materials, experimental systems and methods used in many studies. Here, indicate whether each material, system or method listed is relevant to your study. If you are not sure if a list item applies to your research, read the appropriate section before selecting a response.

Materials & experimental systems

n/a	Involved in the study	
☒	☐	Antibodies
☒	☐	Eukaryotic cell lines
☒	☐	Palaeontology and archaeology
☐	☒	Animals and other organisms
☒	☐	Human research participants
☒	☐	Clinical data
☒	☐	Dual use research of concern

Methods

n/a	Involved in the study	
☐	☒	ChIP-seq
☐	☒	Flow cytometry
☒	☐	MRI-based neuroimaging

Animals and other organisms

Policy information about studies involving animals; ARRIVE guidelines recommended for reporting animal research

Laboratory animals	The mouse strain we used is SwissWebster. Homozygous Tbx1f/f and Tbx1f/f; ROSA26-GFP/GFP mice have been intercrossed and are maintained as inbred lines. Adult breeding mice in our colony range 2-6 months of age. We did not genotype for sex of the embryos; embryos were used randomly depending on genotype.
Wild animals	No wild animals were used
Field-collected samples	No field collected samples were performed
Ethics oversight	All experiments using mice were carried out according to regulatory standards defined by the National Institutes of Health and the Institute for Animal Studies, Albert Einstein College of Medicine (https://www.einsteinmed.org/administration/animal-studies/), IACUC protocol is #0000-1034.

Note that full information on the approval of the study protocol must also be provided in the manuscript.

ChIP-seq

Data deposition

- [x] Confirm that both raw and final processed data have been deposited in a public database such as GEO.
- [x] Confirm that you have deposited or provided access to graph files (e.g. BED files) for the called peaks.

Data access links<br>*May remain private before publication.*	GSE167493, GSE167491, GSE173700 and GSE173521
Files in database submission	GSE167493, GSE167491, GSE173700 and GSE173521
Genome browser session<br>(e.g. UCSC)	NA

Methodology

Replicates	Three replicates were used for TBX1 ChIP-seq.
Sequencing depth	Sequencing depth is provided lines 316-319. We used input as background (sequencing depth replicate 1 is 26,318,633; replicate 2 is 29,821,823; replicate 3 is 33,965,309) to compare with peaks found by ChIP (sequencing depth replicate 1 is 28,070,687; replicate 2 is 32,561,521; replicate 3 is 32,907,512).
Antibodies	We used GFP to sort positive cells by FACS, and we did not use antibodies.
Peak calling parameters	ChIP-seq peaks in each sample were identified using MACS2 2.1.2.1 with default parameters. Then, a consensus list of enriched regions was obtained using the intersectBed function from the BEDTools 2.29 with the default minimum overlap and retaining only the peak regions common to at least two out of the three replicates. Peaks were filtered by removing those overlapping with blacklist regions (Encode mm9 black regions Version 2) using findOverlapsOfPeaks of ChIPpeakAnno.
Data quality	For peak annotation as cis-regulatory regions, GREAT was used with the default settings mm9. The comparison of the gene lists of DAR, DEG and ChIP regions was performed using the dplyr package in R. IGV 2.4.897 was used for peak visualization. Coverage heat-maps and average enrichment profiles (TSS +/- 10Kb) in each experimental condition were obtained using ngs.plot or deepTools2. Significance of the overlap and thereby data quality between list of peaks was evaluated using the ChIPseeker enrichPeakOverlap using mm9 annotation. We applied statistical analysis in processes of all the software and packages described here with default settings.
Software	Cutadapt removes adapter, primers, poly-A tails and it was used to remove unwanted sequence (Martin, https://doi.org/10.14806/ej.17.1.200; https://cutadapt.readthedocs.io/en/stable/)<br>FASTQC (https://www.bioinformatics.babraham.ac.uk/projects/fastqc/),<br>Bowtie2 2.3.4.3 was used to align sequencing reads to reference genomic sequences (Langmead and Salzberg, PMID: 22388286, PMC332281; http://guix.gnu.org/packages/bowtie-2.3.4.3/),<br>MACS2 2.1.2.1 with default parameters was used to identify transcription factor binding sites (Feng et al., PMID: 22936215; PMC3868217, https://pypi.org/project/MACS2/2.1.1.20160226/),<br>BEDTools 2.29 was used to examine genomic intervals from different files (Quinlan and Hall, PMID: 20110278; PMC2832824, https://bedtools.readthedocs.io/en/latest/content/installation.html),<br>dplyr package in R was for data analysis (https://www.rdocumentation.org/packages/dplyr/versions/0.7.8),<br>IGV 2.4.8, was used to visualize data (https://software.broadinstitute.org/software/igv/igvtools),<br>ngs.plot was used to visualize sequencing data (Shen et al., PMID: 24735413; PMC4028082, https://code.google.com/p/ngsplot/),<br>deepTools2 was used for analysis of sequencing data (Ramirez, PMID: 27079975; PMC4987876, https://deeptools.readthedocs.io/en/develop/)

Flow Cytometry

Plots

Confirm that:

- [x] The axis labels state the marker and fluorochrome used (e.g. CD4-FITC).
- [x] The axis scales are clearly visible. Include numbers along axes only for bottom left plot of group (a 'group' is an analysis of identical markers).
- [x] All plots are contour plots with outliers or pseudocolor plots.
- [x] A numerical value for number of cells or percentage (with statistics) is provided.

Methodology

Sample preparation

A numerical value for the number of cells is provided in a new supplementary table, termed Supplementary Table 1. We added a new supplementary figure, Supplementary Fig. 3 that includes further information beyond what is included in Supplementary Fig. 2b, about cell sorting.

Methodology
Sample preparation:
Embryos at E8.0-10.5 were isolated and GFP positive embryos were selected under a SteREO Discovery.V12 microscope (Carl Zeiss, Jena, Germany) in ice-cold DPBS with Ca2+ and Mg2+ (GIBCO, Cat# 14040-133). The rostral half of the embryos were collected at E8.0, E8.25 and E8.5. The pharyngeal apparatus with heart was collected at E9.5 and E10.5. The microdissected tissues were kept on ice, and pooled in DMEM (4 ˚C) until all the dissections were completed. Following centrifugation and removal of DMEM, tissues were incubated with 0.25% Trypsin-EDTA with Dnase I. Then FBS was added to stop the reaction. After centrifugation, the GFP positive cells were resuspended in PBS w/o Ca2+ and Mg2+ with 10% FBS and passed through the 100 µm cell strainer. DAPI was added before cell sorting.

Instrument

The cells were sorted with the BD FACSAria II system (Becton, Dickinson Biosciences).

Software

Data was analyzed with BD FACSDiva 8.0.1 software (Becton, Dickinson Biosciences).

Cell population abundance

We submitted 12,569 cells on average in 11 experiments. These numbers are now provided in new Supplementary Table 1.

Gating strategy

We provide our gating strategy in new Supplementary Figure 3. We show FSC-A/SSC-A (FSC-A, forward scatter area; SSC-A, side scatter area) plot with cells in P1 were gated as cells. The singlets were gated in SSC-H/SSC-W (SSC-height; SSC-width) plot were gated as P2, and shown in FSC-H/FSC-W (FSC-height; FSC-width) plot as P3
and in FSC-A/FSC-H plot as P4, in order. The singlets in P4 gate were plotted to sort GFP positive versus DAPI negative cells.

☒ Tick this box to confirm that a figure exemplifying the gating strategy is provided in the Supplementary Information.
